# Distribution modelling of pre-Columbian California grasslands with soil phytoliths: New insights for prehistoric grassland ecology and restoration

**DOI:** 10.1371/journal.pone.0194315

**Published:** 2018-04-04

**Authors:** Stephen E. Fick, Rand R. Evett

**Affiliations:** 1 Department of Plant Sciences, University of California, Davis, CA, United States of America; 2 Department of ESPM, University of California, Berkeley, CA, United States of America; Ecole Pratique des Hautes Etudes, FRANCE

## Abstract

Historical reconstructions of plant community distributions are useful for biogeographic studies and restoration planning, but the quality of insights gained depends on the depth and reliability of historical information available. For the Central Valley of California, one of the most altered terrestrial ecosystems on the planet, this task is particularly difficult given poor historical documentation and sparse relict assemblages of pre-invasion plant species. Coastal and interior prairies were long assumed to have been dominated by perennial bunchgrasses, but this hypothesis has recently been challenged. We evaluated this hypothesis by creating species distribution models (SDMs) using a novel approach based on the abundance of soil phytoliths (microscopic particles of biogenic silica used as a proxy for long-term grass presence) extracted from soil samples at locations statewide. Modeled historical grass abundance was consistently high along the coast and to a lesser extent in higher elevation foothills surrounding the Central Valley. SDMs found strong associations with mean temperature, temperature variability, and precipitation variability, with higher predicted abundance in regions with cooler, equable temperatures and moderated rainfall, mirroring the pattern for modern perennial grass distribution across the state. The results of this study strongly suggest that the pre-Columbian Central Valley of California was not dominated by grasses. Using soil phytolith data as input for SDMs is a promising new method for predicting the extent of prehistoric grass distributions where alternative historical datasets are lacking.

## Introduction

The invasion of California prairies by Mediterranean grasses and forbs beginning in the 17^th^ century has been one of the most dramatic, yet remarkably undocumented, ecological shifts of the historic era. Very little is known about pre-Columbian vegetation cover on land now characterized as California annual grasslands. Most ecologists assumed that prior to European colonization, grasslands in California were dominated by perennial bunchgrasses, particularly *Stipa pulchra* [1–3]. Clements, who viewed California grasslands as analogous to temperate prairies in the Midwest, proposed that the climax vegetation for California grasslands should be dominated by perennial grasses [1]. He suggested that *Stipa pulchra*, common near roads and railways not exposed to cultivation or grazing, was likely the dominant species of the prehistoric prairie community. Most later researchers continued to assume that large swaths of the Central Valley (Central Valley) were dominated by grasses [2,4,5].

Over the years, however, several examinations of historical accounts and modern relict communities have cast doubt on the assumption that native bunch grasses dominated prehistoric prairies [6–10]. The earliest European explorers’ accounts of vegetation in the Central Valley are sparse and lack species-level detail, but generally suggest either an abundance of flowering broad-leaf plants (forbs) or bare ground, at least in the hot dry summers [8]. Alternative characterizations of early vegetation posit that the majority of California annual grassland sites, particularly in the Central Valley, were prehistorically dominated by annual forbs or shrubs; perennial grasses may have been present but not dominant [11].

Land managers tasked with restoring California 'grassland' sites need solid evidence regarding the prehistoric distribution and abundance of native perennial grasses in vegetation communities in regions currently dominated by California annual grasslands. Pollen records lack the spatial and temporal resolution necessary to provide a detailed understanding of vegetation composition in these areas during the late Holocene [7]. However, a recent large-scale study of soil phytoliths (silt-sized particles of silica formed in and between plants cells) in California provides an opportunity to assess the geographic distribution of sites with long-term prehistoric grass dominance [10]. Although soil phytolith content is influenced by factors such as dissolution, migration via fluvial and aeolian transport, and bioturbation, vegetation composition is likely the dominant driver of soil phytolith content at most locations in California [10]. Grasses are prolific phytolith producers; phytolith content in soils at sites with long-term grass dominance can approach 1–3% of the soil by mass [12,13]. Phytolith content is consistently higher in soil under persistent grassland compared to forests and other vegetation cover, particularly in California where few common plant taxa other than grasses produce abundant phytoliths [13]. Based on accumulation and dissolution rates it is estimated that phytolith assemblages throughout California are the result of at least 1000 years of accumulation [14].

Associations between species occurrence and environmental variables may be used to assess environmental conditions that are suitable for a species (i.e. the niche; [15]). Extrapolation of these relationships across geographic space have been used to predict where conditions are suitable for species occurrence and persistence (e.g. via species distribution models, SDM; [16]). In California, large swaths of grassland have been converted to agriculture, and almost all remnant native 'grassland' has come to be dominated by exotic annual grasses. While relationships between relict patches of native grasses and environmental variables may provide insight into prehistoric distributions at locations that have undergone these disturbances, the phytolith dataset provides a means to test these environment-distribution relationships across a broader geographic range and in a prehistoric context.

Phytolith data collected from a wide geographic range of California annual grassland soil samples indicated a decline in phytolith content when transitioning from coastal to interior California [10]. However, relationships between phytolith assemblages and environmental variables such as climate and soils were not explicitly tested in this study, nor was the potential distribution of pre-Columbian grass-dominated grassland explicitly modeled and mapped.

Our study utilizes a novel approach, for the first time combining an extensive point-based soil phytolith dataset with SDMs to map the estimated prehistoric abundance of a taxon (Poaceae) over a large area (nearly half the state of California) and test assumptions about prehistoric grass dominance. We also examine how soil phytolith content and the modern abundance of native perennial grasses vary with environmental factors, including measures of climate, soil and topography.

## Methods

### Data

**Fig 1:**
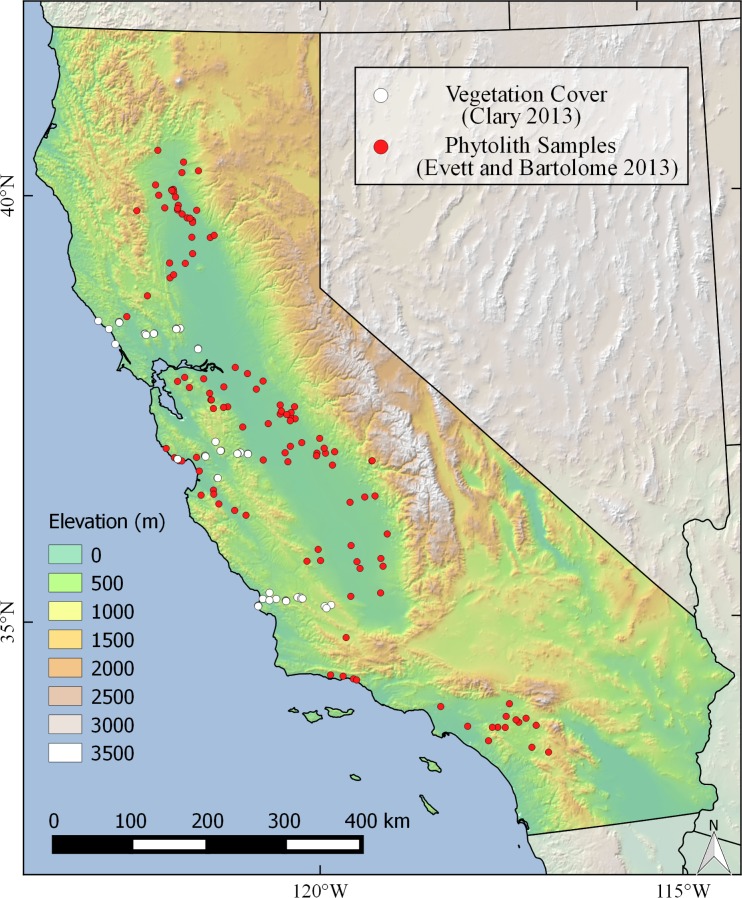
Location of sampling sites used in this study.

We utilized Evett and Bartolome’s [10] soil phytolith content (percent dry weight) data collected from 120 sites throughout non-montane California (Fig 1, S1 Dataset). Phytolith estimates were obtained from the A horizon of a library of statewide soil survey profiles (>2000) collected between 1920–1970, which were screened by location and soil type to avoid soil samples with known biasing effects on phytolith preservation [17,18]. Biasing effects include: 1) soil texture—smaller phytoliths will percolate rapidly downward in coarse-textured soils [19]. Three sandy sites were excluded but a few sandy loam samples were included. 2) soil pH—phytoliths are particularly vulnerable to dissolution in alkaline soils but not in acidic soils [20]. Samples with soil pH > 8.0 were excluded, but soils with pH approaching 8.0 may be more likely to exhibit greater dissolution than acidic soils. 3) differential preservation of morphotypes—thick, blocky morphotypes are better preserved than thin morphotypes [20]. Grass morphotypes are particularly well-preserved in most soil conditions. 4) differential preservation of phytoliths related to soil order–there is evidence that phytoliths are better preserved in Mollisols compared to Alfisols [17]. The majority of the phytolith sites in our study have phytolith content either 50% above or 50% below the 0.30% threshold used by Evett and Bartolome [10], such that even if there were a 50% phytolith content bias in the Alfisol samples compared to Mollisols, for most sites, the phytolith-based classification of a site as long-term grass-dominated would not change.

One major uncertainty in the phytolith data relates to the age of the soil samples. Evett and Bartolome [10] obtained soil samples from the two uppermost A horizons, which varied in depth for each soil, so there was no consistent sampling depth. The upper 60 cm of grassland soils in California is usually well mixed because of intense bioturbation by small mammals (e.g., [21]); this relative uniformity was observed by Evett and Bartolome [10] when they found minimal differences in phytolith content between the A1 and A2 horizons. As explained in previous studies [10, 14], soil grass phytolith content is not a snapshot of one point in time but rather represents a long-term record of grass cover at a site. Using data for current California annual grasslands, the annual phytolith input into the soil is 6 g/m^2^, assuming 75% cover by annual grasses, annual biomass production at 200 g/m^2^ [22], and average grass phytolith production at 4%. Assuming an average soil bulk density of 1.5 g/cc for the upper 60 cm, the weight of the zone of active phytolith mixing is 900,000 g/m^3^. If no phytolith dissolution is assumed, the minimum time required for the soil to reach 0.30% is 450 years. However, research has shown that much of the annual phytolith production is rapidly dissolved in the soil [23], so the actual time required to reach this threshold is likely much longer, probably well over 1,000 years. Because California annual grassland, dominated by exotic grasses, did not arrive in California until less than 250 years ago, soils with phytolith content >0.30% must have been dominated by native grasses for at least several centuries prior to European settlement.

Estimates of modern native perennial grass cover and exotic annual grass cover were obtained from 50 sites in northern and central California ([24]; Fig 1). Climate data included 19 bioclimatic variables derived from monthly climate averages found in the WorldClim dataset, a km-scale interpolated climate surface [25]; these variables are commonly related to biological tolerances for many terrestrial species [26]. An additional climate variable, summertime (May-September) precipitation, was calculated to compare with findings of [24]. Soil data, including bulk density, fractional silt, sand, clay and coarse debris, pH and cation exchange capacity (CEC), were taken from ISRIC SoilGrids 1-km [27]. Estimates of soil texture and chemistry at three depths (0–5 cm, 5–15 cm, 15–30 cm) were integrated to a single value based on weighted average by volume. Approximate wetland area was calculated by aggregating the number of 100 m^2^ grid cells that overlapped at least one wetland feature (e.g., wetlands and vernal pools from the California Aquatic Resource Inventory dataset;[28]) per 1km^2^ (30 arc-second) grid cell on our base map. Slope was derived from the Shuttle Radar Topography Mission (SRTM) 90m spatial resolution ‘hole-filled’ dataset from http://srtm.csi.cgiar.org/.

Pearson moment correlation coefficients were calculated between each environmental variable and soil phytolith content, modern native perennial grass cover, and modern exotic annual grass cover. Because data for modern grass cover estimates were limited to areas within 75 km from the Pacific coast, soil phytolith content was also correlated with environmental variables within this restricted area to facilitate comparison, although there is a noted spatial mismatch at the plot-scale between the datasets.

### Distribution modeling

Numerous techniques exist for modelling species distributions [29]. Model accuracies vary across predictor space and often no single method best approximates distributions under all combinations of environmental attributes [30]. We thus modeled prehistoric and modern native grass-dominated grassland distributions in California using three techniques, then created a final ‘ensemble’ model weighted by cross-validation accuracy.

We used Random Forests, ordinary least squares regression with a lasso penalty (“Lasso”) and generalized additive models (GAM) to model phytolith-environment relationships. Random forests were fit using the R package randomForest [31] with 2000 trees and two randomly selected variables available at each node (mtry = 2). These settings were selected based on consistently low out-of-bag error rates in preliminary analyses. Lasso regressions were fit with the R package glmnet, using optimal cross-validation lambda values obtained from the function cv.glmnet [32]. GAMs were fit with the R package mgcv, with smooth terms for each variable and an extra null-space penalization which effectively removes over-smoothed terms from the model (using the select = TRUE argument). Due to the high number of variables tried relative to data points, only a subset of potential predictors was included for the GAM fits, including distance to coast, annual temperature, temperature isothermality (diurnal / annual range), temperature annual range, annual precipitation, precipitation coefficient of variation, pH, bulk density, sand fraction, and wetland area.

Modeling methods were evaluated using 5-fold cross-validation, and cross-validation correlations between predicted and observed values were used to assign linear weights to individual model predictions (fit with all data) to create the ensemble model prediction. Values for phytolith content were natural log transformed prior to fitting for all models.

The relative importance of environmental variables for explaining phytolith content was evaluated for each model. For Random Forest, the average reduction in prediction error caused by choosing a variable at a split when fitting a decision tree (function varimp) was calculated. For lasso regression, coefficients for models fit on data with predictors scaled to unit variance were used. For GAM, the Chi-square of nested models with each term dropped was used (anova.gam).

Extrapolation to new environments (combinations of environmental variables outside the range of the data set) is problematic for species distribution models [33,34]. To estimate uncertainty due to environmental sampling bias, covariates from 3000 random points across the state were transformed into principal components (PCs) and environmental distance for un-sampled locations was calculated as the average euclidian distance in the first three principle components (comprising > 80% of environmental variation) to the nearest 3 phytolith sampling locations.

Spatial autocorrelation among model residuals (an indication of missing or latent variables and untrustworthy linear model standard errors) was evaluated by comparing observed Moran’s I to 999 bootstrapped simulations using the function moran.mc in the R package spdep [35].

## Results

### Modern perennial grass cover and soil phytolith content correlate with environmental variables

Total soil phytolith content and modern perennial grass cover [24] correlated with most geophysical and climatic variables, in the same direction and to a lesser extent magnitude (Table 1). In general, the associations between perennial grass cover and explanatory variables were stronger than that of phytolith content; however, numerous sites included in the perennial grass study were in a relatively small geographic region (Fig 1), potentially inflating correlations. The magnitude of similarity in correlations between perennial grass cover and phytolith content was generally larger for the subset of phytolith records within 75 km of the coast (the range of data available for perennial grass cover). Modern exotic annual grass cover, in contrast, often correlated with these variables in an opposite direction.

**Table 1:** Environmental correlates.

	Perennial Grass Cover	Phytolith Content (restricted range)	Phytolith Content (full range)	Annual Grass Cover
Slope	0.19	-0.06	-0.04	-0.23
Coast Distance	-0.60***	-0.49***	-0.19*	0.28
Bulk Density	-0.71***	-0.50***	-0.23*	0.30*
CEC	-0.24	-0.35*	-0.19*	0.01
pH	-0.49***	-0.49***	-0.24**	0.1
Coarse Debris	-0.19	-0.02	-0.07	-0.04
Sand	-0.31*	-0.13	-0.03	0
Silt	0.33*	0.30*	0.1	0.01
Clay	0.21	-0.04	-0.05	0.01
Wetland Area	-0.33*	0	0.05	0.25
May-Sept Precipitation	0.40**	0.17	-0.1	0.02
Mean Annual Temperature	-0.54***	-0.35*	-0.26**	0.35*
Mean Diurnal Range (Mean of monthly (max temp—min temp))	-0.69***	-0.44**	-0.30***	0.28*
Isothermality (diurnal/annual range)	0.61***	0.58***	0.34***	-0.42**
Temperature Seasonality (standard deviation)	-0.68***	-0.57***	-0.33***	0.42**
Max Temperature of Warmest Month	-0.72***	-0.57***	-0.35***	0.40**
Min Temperature of Coldest Month	0.61***	0.28	0.22*	-0.26
Temperature Annual Range	-0.73***	-0.56***	-0.35***	0.38**
Mean Temperature of Wettest Quarter	0.51***	0.13	0.02	-0.43**
Mean Temperature of Driest Quarter	-0.70***	-0.53***	-0.34***	0.43**
Mean Temperature of Warmest Quarter	-0.70***	-0.54***	-0.35***	0.41**
Mean Temperature of Coldest Quarter	0.50***	0.19	0.14	-0.35*
Annual Precipitation	0.37**	0.50***	0.13	0.02
Precipitation of Wettest Month	0.35*	0.53***	0.18*	0.06
Precipitation of Driest Month	0.44**	0.18	-0.04	0.01
Precipitation Seasonality (Coefficient of Variation)	0.1	0.24	0.21*	0.03
Precipitation of Wettest Quarter	0.36*	0.51***	0.16	0.03
Precipitation of Driest Quarter	0.39**	0.07	-0.12	0.06
Precipitation of Warmest Quarter	0.71***	0.01	-0.09	-0.50***
Precipitation of Coldest Quarter	0.35*	0.51***	0.17	0.05
Precipitation of Driest Quarter	0.39**	0.07	-0.12	0.06
Precipitation of Warmest Quarter	0.71***	0.01	-0.09	-0.50***
Precipitation of Coldest Quarter	0.35*	0.51***	0.17	0.05

Pearson product-moment correlations between perennial grass cover, annual grass cover, and phytolith content (% dry weight of soil) and geophysical / climatic variables. Phytolith records in “restricted range” are within 75 km of the Pacific Coast (analogous to the distribution of perennial grasses in Clary 2012). Stars indicate significance level (* < = .05, ** < = .01, *** < = .001)

As with perennial grass cover, phytolith content declined considerably as a function of distance from the Pacific Coast. In contrast, modern exotic grass cover increased with distance from the coast, as noted in Clary (2012).

**Fig 2:**
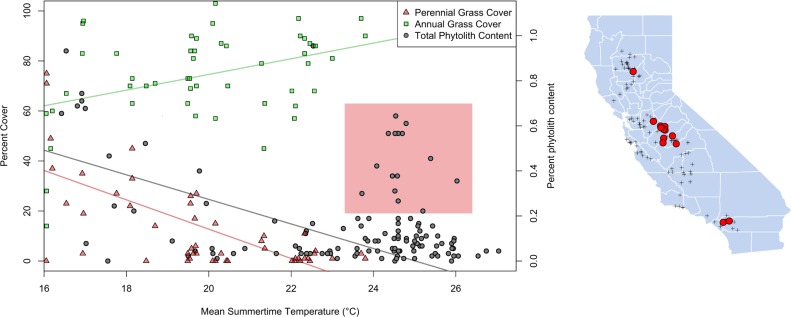
Relationship between mean summertime temperature and modern grass cover. Relationship between mean summertime temperature and modern grass cover [24] as well as phytolith content [10]. Regression line for phytoliths based on data restricted to the region of points from Clary [24]. Points within shaded box are shown at right as red circles against other phytolith samples (black crosses).

Mean temperature of the warmest (summer) quarter was the variable in this study most strongly correlated with annual degree-days above 18°C, identified as a significant explanatory variable for modern grass distribution in previous work ([24] correlation = 0.88, p < 0.001). For phytolith samples less than 75 km from the coast (within the sampling region of Clary 2012), total phytolith content and modern perennial grass cover declined with increasing average summer temperature (Fig 2). Examining the full geographic range of phytolith samples, there was a subset of locations with both high phytolith content and high summer time temperatures clustered in the central Central Valley near the border of Stanislaus and Merced counties (Fig 2 side panel), suggesting there may be additional important environmental variables related to soil moisture, such as depth to water table or amount of fog drip, that were not included in this study. Average May-Sept precipitation was positively correlated with perennial grass cover (r = .40, p = 0.004), but not total phytolith content or annual grass cover (Table 1).

### Distribution models predict higher soil phytolith density along the coast and in the Central Valley foothills than on the floor of the Central Valley

**Fig 3:**
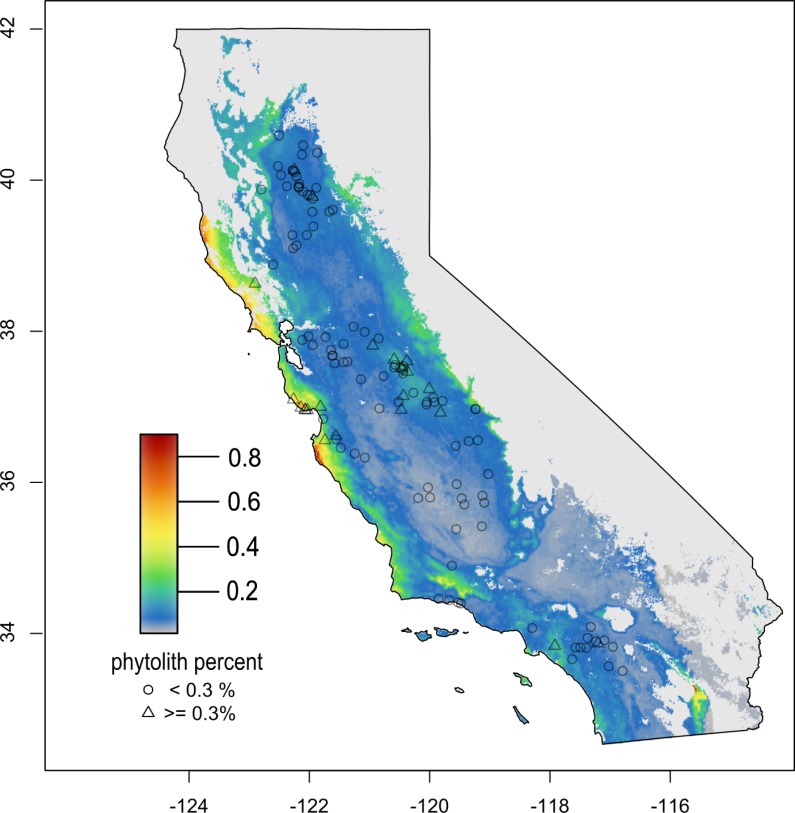
Predicted phytolith abundance. Predicted phytolith abundance in soil (% by mass) for California, based on an ensemble model of three algorithms. Gray region indicates locations > 3 average ‘environmental PC’ units away from the nearest three observed phytolith locations. Circles and triangles represent locations with phytolith content above or below the 0.3% threshold identified by Evett and Bartolome[10].

Areas predicted to have increased soil phytolith content (historical grass cover), based on the ensemble model, were concentrated along the coast and to a lesser extent the Central Valley foothills (Fig 3). There was a band of higher predicted content in the eastern Central Valley foothills, resembling a ‘bathtub ring”, in addition to the area in the northeastern San Joaquin Valley discussed above.

Distribution modeling methods varied in their performance in cross validation, with Random Forest performing the best, followed by GAM and Lasso regression (Table 2). The R^2^ of the ensemble model fit to all data was 0.48, with mean absolute error of 0.097. The mean error for the ensemble model was negative (-0.07) as was the median (-0.002), suggesting that the model tended to under-predict phytolith content on average.

**Table 2:** Cross-validation statistics.

	ρ	R^2^	RMSE	MAE	ME	Moran’s I (p-value)
Random Forest	0.4339	0.1883	0.2	0.12	-0.06	0.94
GAM	0.3958	0.1567	0.2	0.12	-0.05	0.818
Lasso	0.224	0.0502	0.21	0.13	-0.07	0.749

Species distribution model cross-validation statistics, including Pearson correlation coefficient between predicted and actual values (ρ), coefficient of determination (R^2^), root-mean squared error (RMSE), mean absolute error (MAE), mean error (ME) and a test for spatial autocorrelation among residuals (Moran’s I).

**Fig 4:**
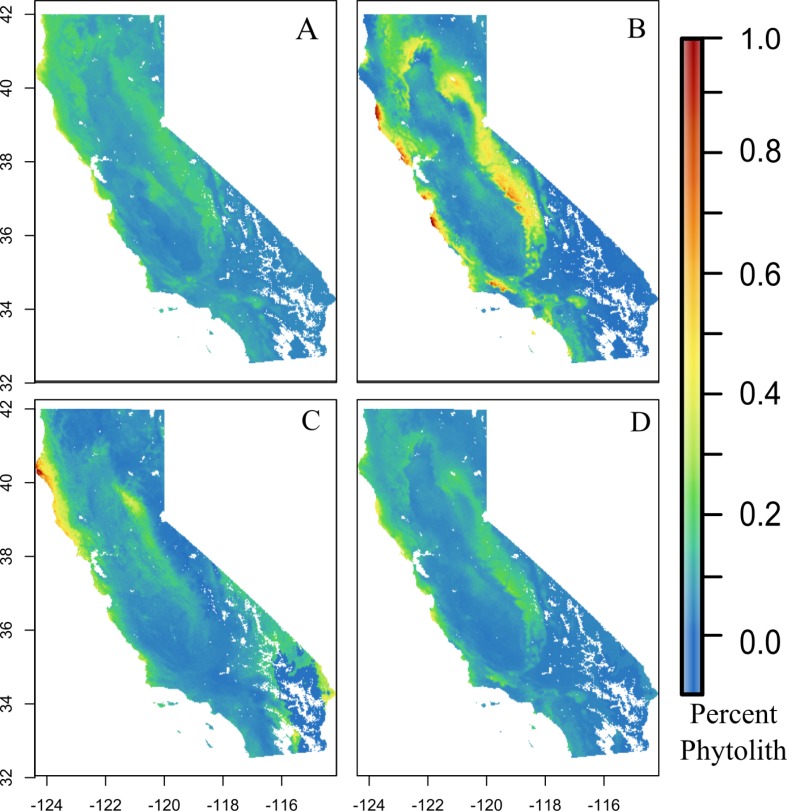
Model predictions. Individual model predictions for soil phytolith content (percent dry weight), and the ensemble model. A) RandomForest, B) GAM, C) Lasso and D) Ensemble model predictions. Note that model predictions are extrapolated beyond the region of confidence (< 3 ‘environmental PC’ units) used in Fig 3. Interior blank areas are regions lacking soils data.

Correlation of residuals between models suggests general similarities between models (all residuals correlation > 0.9). All models captured the trend of high soil phytolith values near the coast compared to interior California, but there were some differences between predictions for the Central Valley and higher elevations in the Sierra Nevada (Fig 4). For example, GAM predicted relatively higher phytolith content for the foothills and coast compared to Random Forest.

The residuals of all models had Moran’s I values not significantly greater than expectation under a null distribution, indicating a lack of detectable spatial autocorrelation in residuals.

### Temperature variation and precipitation seasonality are important distribution model variables

**Fig 5:**
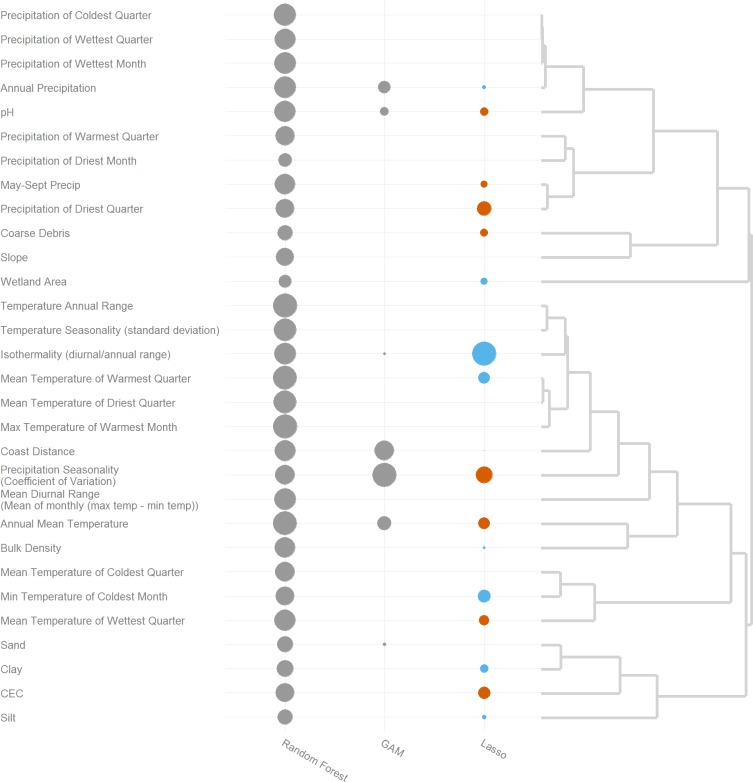
Relative importance of environmental variables for modeling methods. Heuristic comparison of variable importance by SDM model, with size scaled relative to the highest score for each model (score / max score). Dendrogram derived from correlation distance (1 –abs(correlation)) among environmental variables at sampling locations. For tree-based methods (Random Forest and CART), circle size proportional to average increase in node purity. For linear models, circles proportional to scaled coefficients, with red and blue indicating negative and positive coefficients respectively. For GAM, size proportional to X^2^ associated with a reduced model with the corresponding term deleted.

Distribution models selected qualitatively similar groups of variables as important for explaining variation in phytolith content, though they differed in specific variables chosen (Fig 5). Both the Lasso and Random Forest models identified temperature variation as important (group comprised of annual temperature range, temperature of warmest quarter, coast distance and others); the Lasso model indicated higher phytolith content was related to more stable temperatures. Annual mean temperature was also identified as an important predictor. The Lasso and GAM models identified precipitation seasonality as important. Groups of correlated variables related to topography and soil texture (Fig 5, bottom cluster), and minimum temperatures were not assigned much relative importance by models.

Predicted phytolith content generally increased with average calculated NPP. However, when this trend was removed from SDM results, coastal regions continued to have higher predicted phytolith content than the Central Valley interior (S1 Appendix, S1 Fig). Lower foothills of the northern Central Valley had lower predicted phytolith contents than expected given a linear NPP-phytolith content relationship.

**Fig 6:**
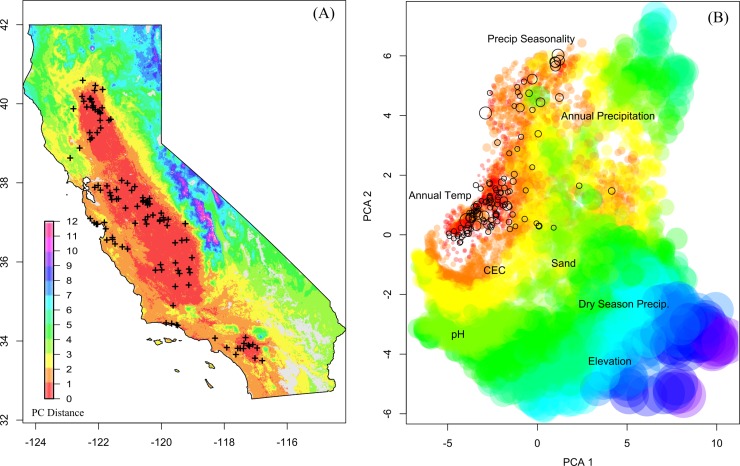
Relative importance of environmental variables for modeling methods. (A) Environmental PC distance from sampling locations mapped across the state. ‘Cooler’ colors represent locations that are environmentally distant from the phytolith sample locations. (B) First two principle components of environmental predictors used in this analysis across the state of California. Unfilled circles represent phytolith locations, with size proportional to phytolith content. Filled circles represent regular samples from across the state, with size and color proportional to Euclidean distance for the first 3 PCs (comprising >80% of the variance) from phytolith locations. Positions of text indicate proportional variable loadings on first two PCs.

Confidence in the species distribution model estimates, based on the density of observations under specific combinations of environmental conditions, was highest in the Central Valley and Los Angeles basin, followed by the central and southern coasts (Fig 6A). Support was high in these regions even where geographic density of samples was low (e.g. the central Sacramento Valley). Environmental conditions were less analogous in the northern and eastern parts of the state, including the north coast and Sierra Nevada. These differences were likely related to quantity and seasonality of precipitation as well as temperature (Fig 6B)

## Discussion

### Evidence for minimal grass dominance in the Central Valley

The ensemble species distribution model predicts low phytolith content throughout most of the Central Valley, with increased abundance along the coast and at slightly higher elevations in the foothills (particularly the eastern Sierra foothills, Fig 3). A similar pattern was found with models that focused on bilobate phytoliths, a morphotype which in California is found only in native grasses *Stipa (Nassella)* and *Danthonia*, two genera that were likely important components of a pre-Columbian native perennial grass-dominated grassland ([10]; S2 Appendix, S2 Fig). Based on this phytolith evidence and available environmental data, it is unlikely that California’s Central Valley prehistorically had persistent, extensive grass cover. Grass-dominated vegetation in the Central Valley may have occurred in relatively small regions where climatic and/or edaphic conditions were suitable.

One anomalous region, located roughly between Merced and Fresno in the central-eastern San Joaquin Valley, was predicted to have relatively high soil phytolith content by the SDMs. This is because several soil samples from this area contained unexpectedly high phytolith content, given climate and distance from the coast (Fig 2). The soils of this region, known as the ‘Sandy Mush’ country, commonly have high sand fractions derived from the glacial erosion in what is now Yosemite National Park [36]. Sandy soils in this region may favor deep-rooted bunchgrasses due to deep percolation of water; more clay-dominated soils in the rest of the valley may favor shallow rooted annual species [36]. An alternative explanation is that most of the sites with high phytolith content are near rivers and may have an elevated water table and/or greater tree cover, maintaining enough soil moisture throughout the dry season to support perennial grasses.

Although the distribution of phytolith sampling locations was not uniform throughout the Central Valley or along the coast, many un-sampled locations within these areas are environmentally similar to locations that were sampled (Fig 6A), increasing confidence in species distribution model predictions. Relative to the entire range of variability in environmental conditions in California, the region of high confidence was fairly limited, with increased uncertainty at higher elevations and latitudes. Distribution models generally predicted higher phytolith contents in high altitude, forested locations, but soils from sites not currently covered by California annual grasslands were excluded from sampling in Evett and Bartolome’s [10] study. In the absence of competition from trees and shrubs, these locations may indeed be suitable for native perennial grasses, exemplified by interstitial meadows in the Sierra Nevada and northern coastal range.

### Environmental controls on prehistoric and historic grass distribution

Soil phytolith content and inferred prehistoric grass cover appear to be driven largely by temperature, particularly temperature extremes and temperature stability, a pattern also found for modern perennial grass assemblages [24], which were strongly (negatively) correlated with temperature range and maximum temperature of the warmest month (Table 1). These temperature variables are strongly correlated with distance from the coast (Fig 5), a single variable which integrates much of this climatic variation. *Stipa (Nassella) pulchra* and most other putative dominant grasses in prehistoric California prairies share a C_3_ ("cool season") photosynthetic pathway, which is favored under cooler, moist conditions. Along the coast, where native perennial grasses continue to be competitive and often dominant [24,37], cooler temperatures and summer input of moisture via fog likely give these grasses an advantage over exotic annual grasses and forbs. Further inland, where temperature variations are more extreme, this advantage is diminished, especially when annual grass and forb species deplete shallow soil water resources early in the growing season [38].

Species distribution model variable importance scores and environmental correlations both indicate precipitation is less important for explaining distribution of phytolith abundance than temperature (except for the GAM), supporting the weak relationship between modern perennial grass cover and summer precipitation noted by Clary[24]. Throughout non-montane western California, precipitation is strongly seasonal and almost uniformly Mediterranean (very low summer precipitation), in contrast to the Iberian peninsula, where summer precipitation was identified as a key factor determining the relative dominance of perennial vs. annual grasses [39]. Perennial grasses in California apparently need a non-precipitation source of moisture during the summer to survive the summer drought. Fog drip fulfills this need along the coast, and an elevated water table is likely required in the Central Valley and surrounding foothills. Topographic and edaphic factors were generally not identified as important drivers of phytolith distributions according to SDM variable importance scores (with the possible exception of pH). Compared to climate variables, important variation in soil and topographic indices can occur on small spatial scales (10–100 m), much smaller than the resolution of data used in this study (1km for soils, 90 m for topography). However, using values imputed directly from soil taxonomy data, Evett and Bartolome [10, supplemental material] found no significant correlations between phytolith abundance and soil texture or chemistry. This may alternatively be viewed as a lack of explanatory importance for these variables, a confirmation that soil properties did not bias the preservation of phytoliths, or as an artefact of a resolution mismatch between soil and phytolith data.

### Conclusions and implications

Our analysis of modern native perennial grass cover and prehistoric soil phytolith distribution strongly suggests that the pre-Columbian Central Valley of California was not dominated by native perennial bunchgrasses, at least to the extent envisioned by early ecologists and many land managers today attempting to restore vegetation to pre-European settlement conditions. Plant communities dominated by native bunchgrass were likely confined to regions with cooler summer temperatures, generally occurring along the coast and at (even slightly) higher elevations. Although undoubtedly widely present in the Central Valley, perennial grasses were likely only locally dominant in restricted areas associated with elevated water tables.

The ranges of climatic and environmental variables found in the Central Valley and much of coastal California were well-sampled by the soil phytolith locations in this study, lending confidence to species distribution model predictions in these regions. More soil phytolith data, sampled across the diverse range of ecological conditions found throughout the state are necessary to resolve uncertainties in grass distribution around the periphery of well-sampled locations.

There is a growing interest in the introduction of diverse, robust assemblages of native annual grasses and forbs for California prairie restoration [9]. Technical research and capacity building for restoration has focused primarily on perennial grasses such as *Stipa (Nassella) pulchra*, but there is increasing awareness of the need for research exploring the dynamics of native forbs [40,41]. When land management goals seek to restore pre-Columbian native species assemblages to California prairies, our study indicates that non-grass species should be emphasized in most of the Central Valley.

An accurate depiction of the historical distribution of ecological communities should be a critical component for designing targets for conservation and restoration interventions [42]. This study demonstrates how soil phytoliths, an underutilized source of data for the reconstruction of historical species distributions, can be combined with modern species distribution models to improve our understanding of historical baselines. Given an appropriate sampling scheme, this methodology can be applied to both grassland and non-grassland contexts where historical documentation of the extent of phytolith-producing species is limited or unavailable at the required spatial scale.

## Supporting information

S1 Fig Phytolith content predictions relative to NPP. Top right inset shows relationship between phytolith content predictions and NPP, as well as fitted line (red). Main plot shows difference between SDM phytolith predictions and NPP-calibrated predictions (i.e. residuals from trend line in inset). Coastal regions have higher phytolith contents than would be expected based on NPP alone (or inversely, Central Valley contents are lower). The foothills also have lower predicted phytolith contents than would be expected based on NPP.
(PNG)

S2 Fig Predicted concentrations of bilobate phytoliths. Predicted concentrations of bilobate phytoliths, diagnostic for species of the genera *Stipa* and *Danthonia*, per g soil, based on ensembled SDM.
(PNG)

S1 Appendix Calibration of phytolith densities by regional productivity. (DOCX)

S2 Appendix SDMs of bilobate phytolith densities. (DOCX)

S1 Dataset Original phytolith density data. (XLSX)
