# Supplementary figures and images for: Distribution modelling of pre-Columbian California grasslands with soil phytoliths: New insights for prehistoric grassland ecology and restoration
Source: PLoS One. 2018 Apr 4;13(4):e0194315. doi: 10.1371/journal.pone.0194315 (PMC5884503; doi:10.1371/journal.pone.0194315)

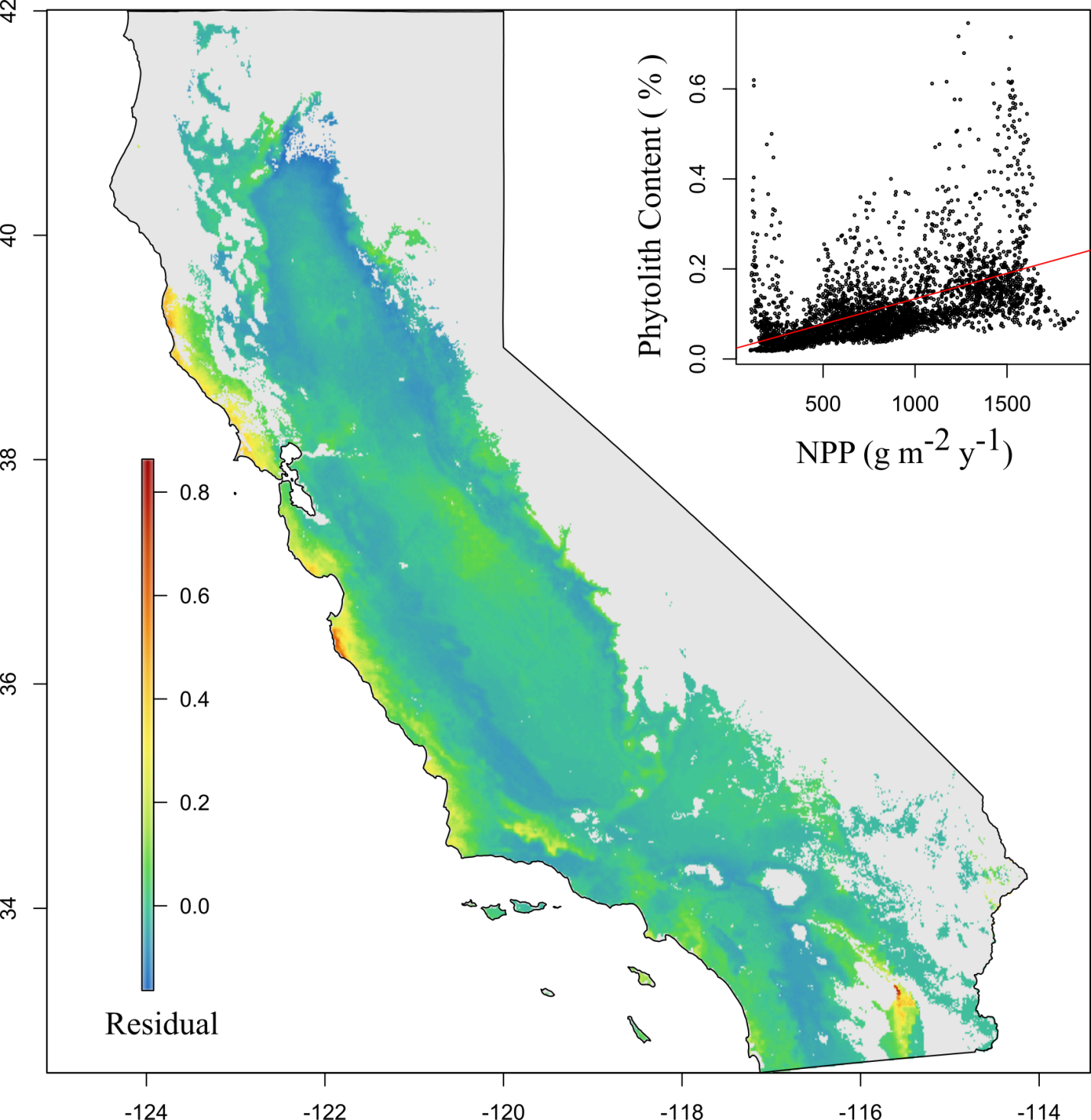

Supplement: S1 Fig — Top right inset shows relationship between phytolith content predictions and NPP, as well as fitted line (red). Main plot shows difference between SDM phytolith predictions and NPP-calibrated predictions (i.e. residuals from trend line in inset). Coastal regions have higher phytolith contents than would be expected based on NPP alone (or inversely, Central Valley contents are lower). The foothills also have lower predicted phytolith contents than would be expected based on NPP. (PNG) [file pone.0194315.s001.png]

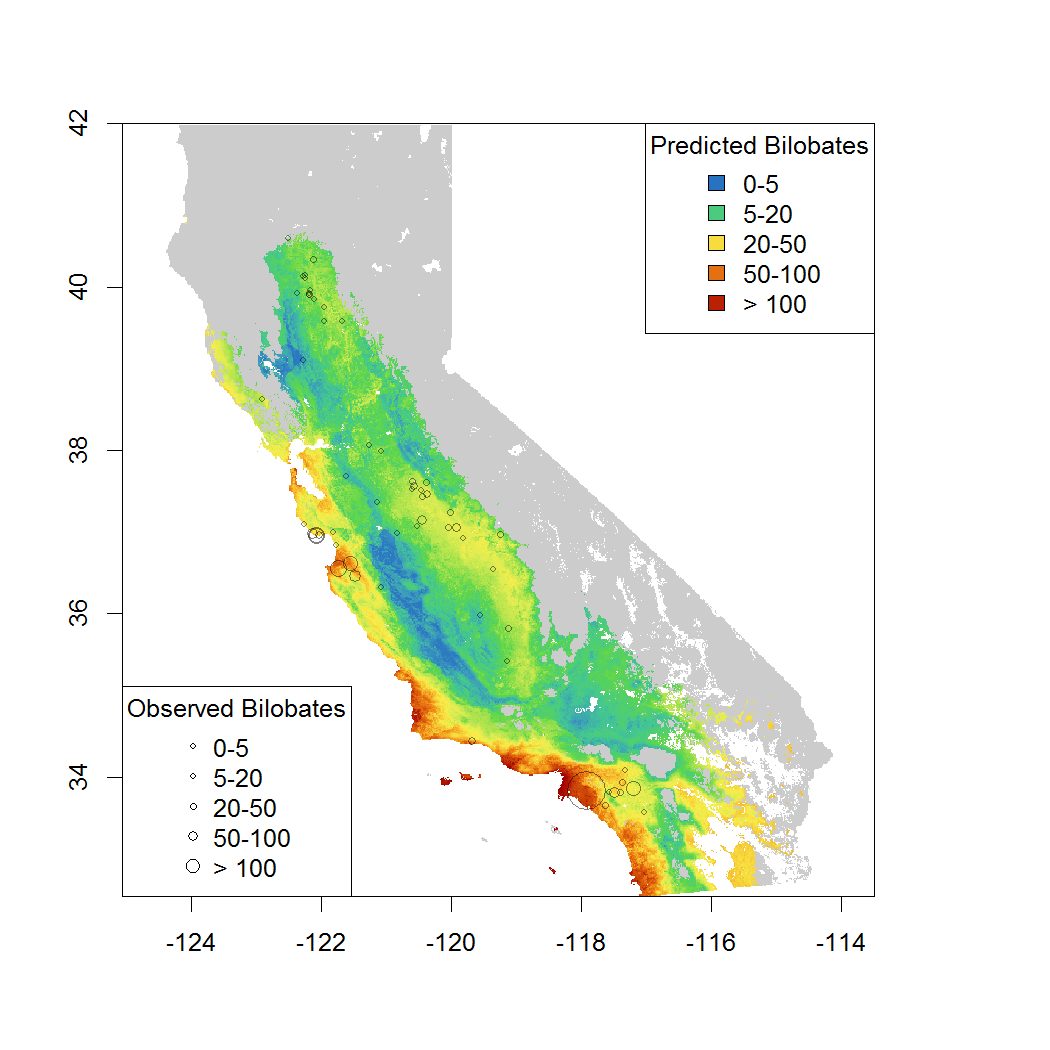

Supplement: S2 Fig — Predicted concentrations of bilobate phytoliths, diagnostic for species of the genera Stipa and Danthonia, per g soil, based on ensembled SDM. (PNG) [file pone.0194315.s002.png]
