# Supplementary material for: Distribution modelling of pre-Columbian California grasslands with soil phytoliths: New insights for prehistoric grassland ecology and restoration
Source: PLoS One. 2018 Apr 4;13(4):e0194315. doi: 10.1371/journal.pone.0194315 (PMC5884503; doi:10.1371/journal.pone.0194315)
Supplement: S1 Appendix — (DOCX) [file pone.0194315.s003.docx]

***S1 Appendix : Calibration of phytolith densities by regional productivity***

To evaluate whether differences in phytolith density might simply be the result of differences in primary productivity at different locations (e.g., low grass signal in the San Joaquin valley due to low overall biomass there) we calibrated model predictions by approximate net primary productivity (NPP). We fit a linear relationship between model predictions and expected NPP, then subtracted this effect from predicted surfaces. We calculated NPP using the ‘Miami Equation’ from Lieth (1975) and Worldclim climate surfaces (Hijmans et al. 2005):

$$NPP=\min(NPPt, NPPp)$$

With

$$NPPt={3000 \left( 1+\exp\left( 1.315-0.119*\bar{T} \right) \right)}^{-1}$$

And

$$NPPp=3000 \left( 1-\exp\left( -0.000664*\bar{P} \right) \right).$$

Where $\bar{T}$ and $\bar{P}$ are mean annual temperature and annual precipitation respectively, NPPt is temperature-limited NPP and NPPp is precipitation-limited NPP in g dry matter / m^2^ / y.

*Results*

See S1 Figure.

*Discussion*

The argument that differences in phytolith content between coastal and interior California is due to coastal regions being more productive (in the absence of irrigation), leading to higher biomass and higher phytolith content in coastal soils despite similar grass cover, was not supported by the data. After correcting for the potential (linear) effect of NPP on phytolith content, large differences between the coastal and inland regions remained, indicating that higher content along the coasts is probably not due to differences in potential productivity alone.

*Refrences*

Hijmans, R. J., S. E. Cameron, J. L. Parra, P. G. Jones, and A. Jarvis. 2005. Very high resolution interpolated climate surfaces for global land areas. International Journal of Climatology 25:1965–1978.

Lieth, H. 1975. Modeling the primary productivity of the world. Pages 237–263 *in* H. Lieth and R. H. Whittaker, editors. Primary productivity of the biosphere. Springer, Berlin.
