# Supplementary material for: Distribution modelling of pre-Columbian California grasslands with soil phytoliths: New insights for prehistoric grassland ecology and restoration
Source: PLoS One. 2018 Apr 4;13(4):e0194315. doi: 10.1371/journal.pone.0194315 (PMC5884503; doi:10.1371/journal.pone.0194315)
Supplement: S2 Appendix — (DOCX) [file pone.0194315.s004.docx]

***S2 Appendix: SDMs of bilobate phytolith densities***

The distribution of bilobate phytoliths, which are diagnostic for species of the genera *Stipa* and *Danthonia* in California, the putative dominant bunchgrasses of coastal regions and interior valleys were analyzed in the same manner as that of overall phytolith abundance in the main text. Data used for this analysis included the number of bilobate phytoliths per g of soil. Only half of the samples in Evett and Bartolome (2013) contained any bilobate phytoliths, so models of bilobate density were restricted to these records. Additionally, due to the limited number of data points with bilobate information (60 out of 120) GAMs of bilobate content utilized smooths of coast distance, the first principle component of a PCA using only climate variables and the first principal component of a PCA only using soil and topographic variables.

*Results and Discussion*

Species distribution models for bilobate phytoliths, indicative of grass species in the genera *Stipa* and *Danthonia*, showed similar patterns to that of SDMs of overall phytolith density. The highest predicted bilobate abundance occurred along the southern coast of California, near Santa Barbara and the Los Angeles basin, as well as the Salinas valley (S2 Fig). Bilobate content was predicted moderately low throughout the CV, with higher bilobate density found in northeastern San Joaquin Valley. Modeling methods tended to perform poorly in 5-fold cross-validation, perhaps because only half of the samples contained any bilobates, with average R^2^ ranging from 0.004 to 0.05. Final model R^2^s (fit to all data) for bilobate content was 0.79.

Bilobate phytoliths were present throughout the sampling range, but inconsistently so, with only half of the collected samples containing any bilobate phytoliths. The only consistent trend in bilobate prevalence revealed by the SDMs was increased absolute bilobate abundance as well as relative abundance (compared to other phytoliths) along the central and southern coasts, supporting the hypothesis that native perennial bunchgrasses such as *Stipa pulchra* were likely abundant primarily in coastal regions and perhaps in scattered patches within the Central Valley.

It is noted that in the floristically diverse state of California there are numerous native species of true bilobate-producing panicoid grasses -- however these are only found in warm, interior areas of the state that remain moist year around. Only a very small percentage of non-irrigated land in California fits this description, exclusive of the areas sampled in this study ( see Evett et. al 2012 for details.)

|  | **cor** | **R2** | **rmse** | **MAE** | **ME** | **Median** | **I** |
| --- | --- | --- | --- | --- | --- | --- | --- |
| rf.statistic | 0.1972 | 0.0389 | 47.18 | 15.82 | -11.44 | 0.79 | 0.999 |
| gam.statistic | 0.0587 | 0.0034 | 50.94 | 18.72 | -7.76 | 0.02 | 0.9988 |
| lasso.statistic | 0.2391 | 0.0572 | 47.44 | 15.81 | -12.3 | 0.72 | 0.9988 |

Table S1. Cross validation statistics for bilobate content.

*References*

Evett, R. R., and J. W. Bartolome. 2013. Phytolith evidence for the extent and nature of prehistoric Californian grasslands. The Holocene 23:1644–1649.
